# Introduction: frontiers of applied inverse problems in science and engineering

**DOI:** 10.1098/rsta.2024.0056

**Published:** 2025-09-25

**Authors:** Danny Smyl, Andreas Hauptmann, Tyler Tallman

**Affiliations:** ^1^ School of Civil and Environmental Engineering, Georgia Institute of Technology, Atlanta, GA, USA; ^2^ Research Unit of Mathematical Sciences, University of Oulu, Oulu, Finland; ^3^ Department of Computer Science, University College London, London, UK; ^4^ School of Aeronautics and Astronautics, Purdue University, West Lafayette, IN, USA

**Keywords:** inverse problems, machine learning, civil engineering, aerospace engineering, structural engineering

## 1. Introduction

Inverse problems have always been about peering through a mirror—looking backwards from the things we can measure to learn about the things we cannot directly see. In the last few years, that mirror has been polished by faster algorithms, smarter statistics and a new ally in machine learning. In the recent decades, it became apparent that inverse problems are not limited to the classical mathematical study of inverse boundary value problems, such as the Calderón problem [1], but have widespread practical applications. In fact, many scientific and engineering problems can be studied under the lens of inverse problems, revealing new viewpoints and enabling a new suite of methods [2,3]. Motivated by this insight, we edited this special issue on the *frontiers of applied inverse problems in science and engineering*, collecting thirteen papers, which were chosen to show how far the field has come and, more importantly, where it may be heading next. This special issue illustrates these advances by specifically highlighting how inverse problems are relevant and making an impact in a wide range of scientific fields, from aerospace and civil engineering to medical imaging, benefitting society and health at large. Each individual article tackles a practical question that used to feel out of reach and answers it with a blend of mathematical rigour and computational daring.

Chung and co-authors show how to tame one of the biggest computational bottlenecks in Bayesian inference: picking the correct hyper-parameters. Their sample-average approximation turns what was once an art form into something approaching push-button automation, making fully probabilistic inversions feasible on everyday hardware. Zhang and colleagues push deeper into operator learning with their coefficient-to-basis network, a nimble architecture that changes its own resolution as it learns—a fine-tunability that could become a template for future solvers. Worden, Rogers and Preston return to the venerable Volterra series, refreshing it with modern identification tricks and reminding us that ‘classical’ does not have to mean ‘dated’. Calvetti’s team keeps physiology front and centre by applying separable hierarchical priors to extract and analyse muscle synergies in human locomotion, embedding physiological structure directly into their under-determined biomechanics models.

If data are the new currency, material scientists are hitting the jackpot. Childs and collaborators use Bayesian machine learning to design ultra-high-performance concrete mixes that are known to be tedious to uncover by trial and error. Tian and co-authors’ learning latent hardening framework folds micromechanical knowledge straight into a neural network, proving that black-box models can carry real metallurgical insight inside. Meanwhile, Esteghamati takes a similar philosophy into earthquake engineering, crafting an explainable surrogate that turns the once-esoteric loop of performance-based seismic design into an engineer-friendly inverse problem.

Imaging, of course, is commonly considered the public face of inverse problems. Park, Jeon and Seo critically examine the mathematical foundations of low-dose, cost-effective dental cone beam computed tomograph—identifying research directions to enhance image quality by tackling metal-induced artefacts via alternative forward-model formulations beyond the Radon transform and by assessing the promise and limitations of deep learning-based methods. Ashraf and co-authors boost positron emission tomography reconstructions with an elegant deep-image prior, rescuing signal from the faintest of low-count acquisitions. Mueller demonstrates that electrical impedance tomography can pick up a newborn’s ventilation and perfusion without radiating the infant—proof that gentle medicine and hard mathematics can coexist. Homa’s group tackles the industrial end of the spectrum, segmenting eddy-current data with matching component analysis so that tiny defects in aerospace alloys no longer slip through the net. Nguyen and Le close the imaging loop with an orthogonality-sampling method for Maxwell’s equations that works not just on simulations but on noisy experimental data.

Finally, Karlsen and colleagues remind us that inverse problems can be explosive—literally. Their trilateration scheme infers both the yield and the point of origin of a blast wave from sparse pressure readings, offering a rapid forensic tool that could one day guide emergency response teams in real time.

Taken together, the articles in this special issue deliver on our original intent: to illustrate how state-of-the-art mathematics, judicious statistics and physics-savvy machine learning are converging into a single theme. They lower computational barriers, widen the range of data we can trust and move several applications from ‘promising’ to ‘practical’. Just as importantly, they flag the obstacles still ahead—from scaling to exascale hardware to keeping models honest when data are scarce. We hope the reader comes away energized, convinced that the next decade of inverse problems will be not only about seeing the unseen but about turning that vision into safer structures, stronger materials, cleaner images and, ultimately, better decisions.

## Data Availability

This article has no additional data.
